# Plant polysaccharides influence tumor development based on epigenetics: a review

**DOI:** 10.3389/fphar.2025.1588857

**Published:** 2025-05-06

**Authors:** Cong Li, Yuao Hu, Hua Yang

**Affiliations:** ^1^ College of Bioscience and Biotechnology, Hunan Agricultural University, Changsha, China; ^2^ College of Food Science and Technology, Hunan Agricultural University, Changsha, China

**Keywords:** plant polysaccharides, epigenetic, tumor development, oncology agent, apoptosis

## Abstract

Plant polysaccharides have emerged as pivotal epigenetic modulators in oncology, offering multi-target therapeutic potential to address toxicity and drug resistance limitations of conventional therapies. This review integrates evidences from multi-database (PubMed, Web of Science and CNKI, 2010–2025) to elucidate three core epigenetic mechanisms of plant polysaccharides (e.g., Astragalus and Ganoderma lucidum): 1) TET2-mediated DNA demethylation; 2) inhibition of histone-modifying enzymes including JMJD2D; 3) regulation of tumor-suppressive miRNAs such as miR-139-5p. Preclinical studies demonstrate synergistic effects with chemotherapeutics, enhancing antitumor efficacy while reducing toxicity through immune modulation (e.g., H22 murine models) and organ protection (e.g., cisplatin regimens). Bibliometric analyses further uncover emerging roles in tumor microenvironment reprogramming, angiogenesis suppression, and macrophage polarization. These findings establish plant polysaccharides as precision oncology agents bridging molecular mechanisms with clinical translation. Future research should prioritize structural standardization, pharmacokinetic profiling, and combinatorial therapy optimization to accelerate clinical translation.

## 1 Introduction

Tumor is a disease caused by gene mutation, leading to irreversible and unlimited proliferation of cells, and it is characterized by high incidence and high mortality (Yang et al., 2022). It is estimated that by 2030, the occurrence of malignant tumors worldwide will lead to 13 million deaths (Abu-Khudir et al., 2020) Current therapeutic strategies, including chemotherapy and surgical resection, are often limited by severe side effects, drug resistance, and irreversible damage to healthy tissues (Roma-Rodrigues et al., 2019). These challenges underscore the urgent need to find safer, more effective therapeutic alternatives.

Plant polysaccharides, as natural bioactive macromolecules, have emerged as promising candidates for tumor therapy due to their multi-target mechanisms, low toxicity, and ability to synergistically modulate key pathways in tumorigenesis. Unlike conventional therapies, polysaccharides exhibit pleiotropic effects that align with the evolving paradigm of multi-targeted cancer treatment. Their anti-tumor efficacy is mediated through diverse biological activities, including direct inhibition of tumor cell proliferation (Sohretoglu and Huang, 2018), induction of apoptosis (Zhang et al., 2024), suppression of metastasis (Hu et al., 2024) and immunomodulation (Wei et al., 2019). Critically, polysaccharides have been shown to possess the capacity to overcome drug resistance, a major limitation of chemotherapy, by targeting epigenetic regulators and tumor microenvironment components (Nagaraj et al., 2016; Feng et al., 2018).

Epigenetic dysregulation, including aberrant DNA methylation, histone modifications, and non-coding RNA expression, is increasingly recognized as a hallmark of tumor initiation and progression (Ciela, 2024; Liu et al., 2024; Wei et al., 2024). Notably, nearly half of transcriptional changes in cancer driver genes are linked to mutations in epigenetic regulators (Nagaraj et al., 2016), highlighting the therapeutic potential of agents that restore epigenetic homeostasis.

Plant polysaccharides have unique mechanisms to regulate epigenetic homeostasis: for example, Dendrobium polysaccharides enhance TET2-mediated DNA demethylation (Mirzadeh et al., 2021), while Ganoderma lucidum polysaccharides regulate histone acetylation via H3K27ac signaling (Elsawi et al., 2022). Such epigenetic reprogramming can reverse tumor-promoting gene expression patterns without inducing genotoxic stress, offering a strategic advantage over DNA-damaging chemotherapeutics.

Furthermore, polysaccharides exhibit synergistic effects with existing therapies, thereby mitigating toxicity and enhancing efficacy. Preclinical studies demonstrate that polysaccharides can protect organs from chemotherapy-induced damage (Jiao et al., 2016; Li et al., 2024), improve immune function (Jen et al., 2021), and inhibit angiogenesis (Gong et al., 2022), thereby overcoming the dual challenges of therapeutic resistance and systemic toxicity. The structural diversity of polysaccharides, influenced by molecular weight, glycosidic linkages, and monosaccharide composition, enables tailored interactions with multiple cellular targets (Barbosa and Junior, 2020), and provides a versatile platform for drug development.

This review systematically evaluates the epigenetic mechanisms underlying the anti-tumor effects of plant polysaccharides. A comprehensive literature search was conducted using PubMed, Web of Science and CNKI databases (2010–2025) with keywords 142 studies meeting quality thresholds (e.g., robust experimental design, statistical validation) were selected for critical analysis. By integrating evidence from mechanistic studies and clinical observations, this review highlights the translational potential of plant polysaccharides as epigenetic modulators in precision oncology.

## 2 Methodology

This systematic review comprehensively investigated the epigenetic mechanisms of plant polysaccharides in antitumor therapy through a structured methodological approach. A multi-database search spanning PubMed, Web of Science, and CNKI (January 2010 to April 2025) was conducted using Boolean operators combining controlled descriptors (“plant polysaccharides,” “epigenetic regulation”) with free terms (“DNA hydroxymethylation,” “lncRNA networks,” “chemosensitization”). The initial 387 identified records underwent rigorous triage: 125 duplicates were removed via EndNote X9 and manual verification, followed by two-phase screening by independent researchers applying predefined eligibility criteria. Inclusion required peer-reviewed studies demonstrating polysaccharide-induced epigenetic reprogramming in tumor models (cell lines/animal studies) or clinical combination regimens, while excluding non-mechanistic analyses and non-polysaccharide phytochemical research. From 262 full-text assessments, 120 studies (68 preclinical, 42 clinical trials, 10 mechanistic reviews) were retained for in-depth analysis. Data extraction focused on three dimensions: 1) structural attributes including molecular weight distribution (5–2,000 kDa) and heteropolysaccharide configurations (e.g., glucose-mannose-galactose ternary systems); 2) epigenetic regulatory axes encompassing DNMT3A/TET2-mediated DNA methylation dynamics, histone code alterations through JMJD2D inhibition/H3K4me3 modulation, and non-coding RNA circuits regulating miR-139-5p/STAT3 and lncRNA-MALAT1 pathways; 3) translational evidence of chemotherapy synergy, particularly with platinum-based drugs through ABCB1 promoter hypomethylation. Methodological rigor was ensured by evidence-level stratification and cross-validation of pharmacodynamic outcomes against epigenetic biomarker changes (e.g., 5-hydroxymethylcytosine levels correlating with tumor suppression efficacy), establishing robust structure-epigenome-function relationships critical for developing precision phytopharmaceuticals.

## 3 Epigenetic regulation and tumors

### 3.1 The process of tumor formation

The development of malignant tumors is usually a very long process. Existing studies show that the development of malignant tumors usually has the following characteristics. Persistent proliferative signaling is the most basic feature of tumor cells, which will proliferate abnormally under the influence of persistent proliferative signaling. Avoidance of growth inhibitory genes is the second hallmark of tumor development, and tumor cells will undergo immune escape in various ways, for example, specific malignant tumor cells will upregulate expression of tumor antigenic peptide presenting molecules (HMCs) to avoid being subjected to NK-cell-mediated lysis (Bai et al., 2003). In addition, tumors overexpress non-classical MHC-I molecules (e.g., HLA-G) to evade CTL and NK cell-mediated killing, leading to immune escape (Rouas-Freiss et al., 2003); Anti-apoptosis is also an essential feature of tumor development, and tumors usually secrete anti-apoptotic factors (España et al., 2004) or downregulate the expression of Fas receptor on the cell surface (Volkmann et al., 2001; Pedrosa and Fabi, 2024) to resist apoptosis. Induction of angiogenesis is also a hallmark of tumorigenesis and development. High production of angiogenesis-inducing factors, such as VEGF in tumor tissues (Mo et al., 2018), can lead to vascular abnormalities and thus impede the entry of immune cells into tumor tissues (Huang et al., 2018). The above-mentioned features are some of the significant hallmarks of malignant tumor development.

### 3.2 Role of epigenetic regulatory mechanisms in tumorigenesis and development

**FIGURE 1:**
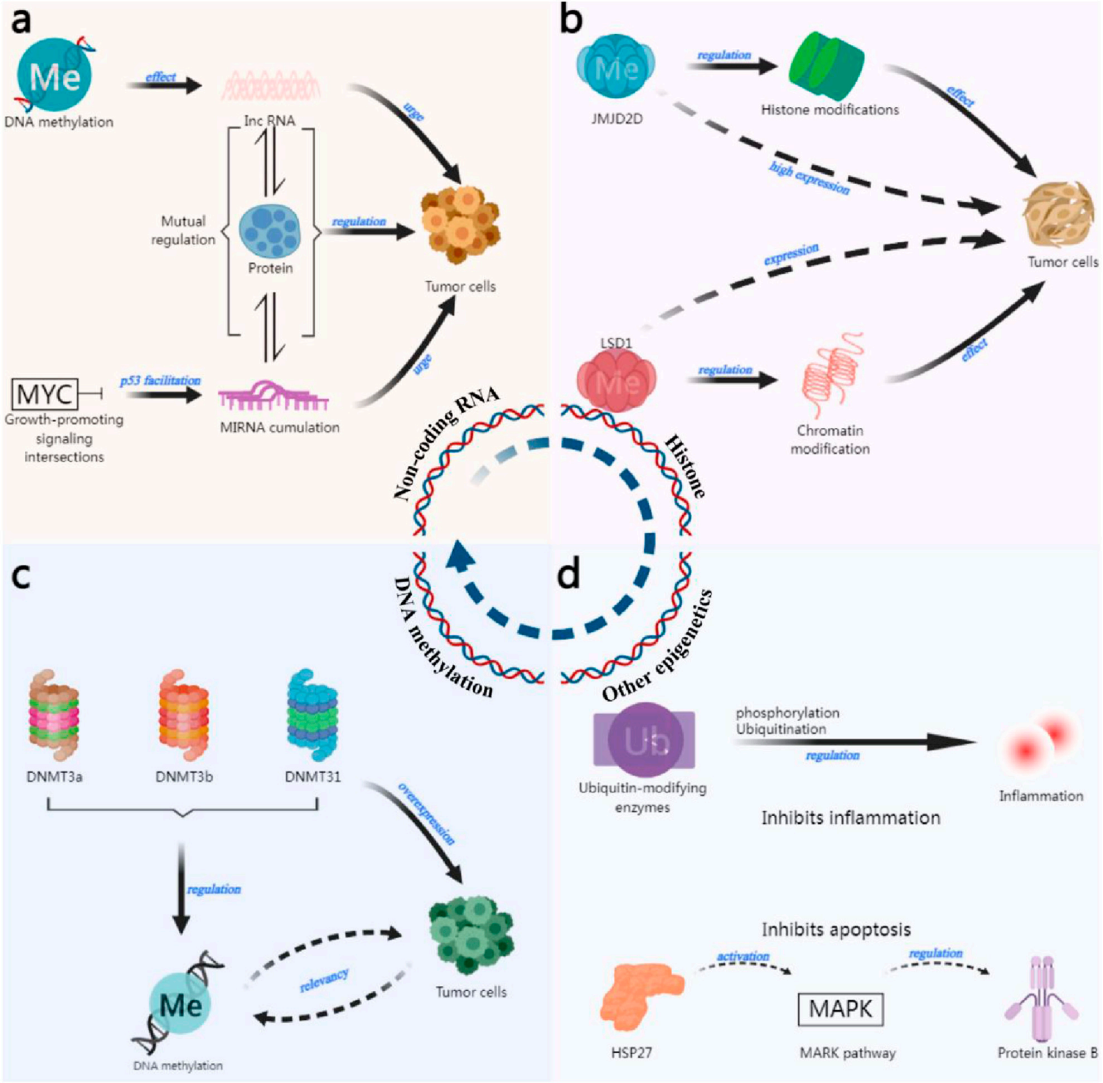
Epigenetic mechanisms of tumor formation. **(a)** The role of non-coding RNAs in tumor development (Dang, 2012; Ritchie et al., 2015; Olivero et al., 2020). **(b)** The role of histone modifications in tumor development (Berry et al., 2014; Yuan et al., 2015; Hu et al., 2018). **(c)** The role of DNA methylation in tumor development (Sakuma et al., 2006; Witt et al., 2009). **(d)** Other epigenetic roles (Li R. J. et al., 2015; Tarcic et al., 2016; Zhao et al., 2020).

Epigenetic inheritance refers to changes that do not alter the DNA sequence but can affect the gene expression and phenotype change and can sometimes be passed on to offspring (Stirzaker et al., 2016), epigenetics mainly includes DNA methylation, histone modification, and non-coding RNA (Egger et al., 2004; Camarena and Wang, 2016) et al. Epigenetic inheritance can also affect the development of individuals and the development of tumors, leading to the malignant transformation of cells into tumors, as shown in Figure 1.

#### 3.2.1 DNA methylation

DNA methylation is the process of attaching a methyl group to the carbon atom at the 5-position of cytosine on the CpG island (cytosine-phosphate-guanine island) catalysed by DNA methyltransferase (DNMT) with S-adenosylmethionine as the donor (Kim and Kaang, 2017). Usually, tumors are abnormal in genome-wide hypomethylation and site-specific hypermethylation (Klutstein et al., 2016). Hypomethylation triggers chromosomal instability and activation of oncogenes or silencing of normal genes (Bakshi et al., 2018). Several studies have shown that DNA methylation can promote the occurrence and development of tumor cells. The early and cumulative events of gallbladder cancer include DNA methylation, and the frequency of methylation increases with the continuous expansion of tumor cells (PreetiSharma et al., 2016). The three transferase enzymes known to regulate DNA methylation in mammals (DNMT3a, DNMT3b, and DNMT31) are usually overexpressed in human tumor cells (Song et al., 2015). A significant increase in DNMT1 levels in bladder cancer tissues has been found to correlate with the hypermethylation of the CpG island promoter of tumor oncogenes (Seo et al., 2017) In addition, some studies have reflected the dual nature of DNA methylation. First, it promotes tumor formation through hypermethylation of oncogenes. Second, it protects genome integrity by inhibiting the translation of repetitive DNA (Tang et al., 2021).

In summary, alterations in DNA methylation can lead to abnormalities in gene structure and function, and can provide an early warning of tumorigenesis (Harsanyi et al., 2022). DNA methylation and the methylation level of specific genes can be used as a diagnostic indicator of the relevant tumor cells.

#### 3.2.2 Histone modifications

Histones usually refer to the basic structures of eukaryotic chromosomes and are highly conserved. Due to slight differences in their amino acid composition; histones are classified into five types: H1, H2A, H2B, H3, and H4. The N-terminal amino acids can be used as modification sites for chemical modifications such as methylation, acetylation, phosphorylation, adenylation, ubiquitination, and adenosine diphosphate ribosylation. Some stem cell studies have shown that the opposite effects of the polycomb group (polycomb proteins are generally associated with gene repression) and the Trithorax group (TRX genes are associated with gene activation) can remodel proteins to regulate many cellular activities, and the development of tumor cells is a very typical example (Chetverina et al., 2017). Hu et al. reported that histone demethylase JMJD2D was highly expressed in gastric cancer, suggesting that the activity of gastric cancer cells causes this methylase to be active (Hu et al., 2018). Berry et al. (2014) also reported a high correlation between the high expression of JMJD2D and the poor prognosis of patients with clear cell carcinoma, which suggests that there is a correlation between the development of tumor cells and histone modification. In lung cancer, the histone enzymes H3 lysine 27 methylase and demethylase are aberrantly expressed, which directly causes histone modification variability. It has been shown that histone demethylase LSD1 is expressed in most squamous cell carcinomas of the tongue, which is closely associated with tumors, and LSD1 can promote tumor cell development through chromatin modification. In contrast, tumor cell activity can be effectively inhibited if LSD1 is reduced in animal models (Yuan et al., 2015). Besides methylation modification, histone acetylation modification (Giudice et al., 2013; Li Q. et al., 2015), histone deacetylation modification (Sakuma et al., 2006; Witt et al., 2009), phosphorylation modification (Lee et al., 2019) and ubiquitination (Huber et al., 2011) are also related to the development of tumor cells. Existing studies revealed close association between various aberrant histone modifications and tumors, and further study of their mechanisms in tumor cells may help elucidate the relationship between histone modifications and tumor activities.

#### 3.2.3 Non-coding RNA

In the human genome, about 70% of the genes are transcribed into RNAs, of which 2% can be transcribed into messenger RNAs (mRNAs), and the rest of the genes are transcribed into non-coding RNAs (ncRNAs) (Anastasiadou et al., 2018). These ncRNAs are roughly divided into two categories. One is the constitutive ncRNAs, including transfer RNA (tRNA), ribosomal RNA (rRNA), etc., which are relatively stable and necessary for cell survival. The other category is regulatory ncRNAs, whose regulation is spatiotemporally or organizationally specific, and which play a role in life activities such as transcription and translation (Lv and Huang, 2019). Regulatory ncRNAs are generally categorised by their length, which is bounded by a length of 200 nucleotides, and those smaller than this value are referred to as small ncRNAs, e.g., microRNAs (microRNAs are also referred to as miRNAs), tRNA-derived small RNAs (tsRNAs), etc. Those larger than this value are referred to as long ncRNAs, such as linear lncRNAs and loop-closing circRNAs (Slack and Chinnaiyan, 2019) In recent years, more and more studies have demonstrated that ncRNAs are associated with tumor development and play important regulatory roles, especially microRNAs, IncRNAs, etc., whose interactions with proteins are highly significant in tumor regulation (Yang et al., 2020; Zhao et al., 2021). IncRNA PVT1, activated by P53-dependent activation, is located 50 kb downstream of the MYC gene (Dang, 2012) (massive growth-promoting signaling crossroads involved in cell proliferation or growth), and is induced by oncogenic signals. PVT1 accumulates near the MYC gene transway point and inhibits its transcription (Olivero et al., 2020), which then affects the proliferation and growth of normal cells and contributes to the further spread of tumor cells. In addition, IncRNA methylation profiles were reinterpreted using a normalized process, and it was found that being affected by DNA methylation also causes IncRNAs to contribute to tumorigenesis (Ritchie et al., 2015). In lung cancer, some miRNAs inhibit the binding of DNMT3A and 3B, and high levels of miR-29b genomic DNA hypomethylation lead to re-expression of oncogenes (Fabbri et al., 2007; Garzon et al., 2009). In summary, it can be found that there will also be mutual regulation among epigenetic regulatory mechanisms, which also have a relationship with tumor cell development.

#### 3.2.4 Other epigenetic regulatory

##### 3.2.4.1 Mechanisms

In addition to the three central epigenetic regulatory mechanisms mentioned above, there are remaining epigenetic regulatory mechanisms that have gradually become hot research issues in the field in recent years, such as phosphorylation, ubiquitination, and other epigenetic mechanisms, whose unique mechanistic expression is also an indispensable part of the tumor development process. It has been reported that ubiquitin-modifying enzymes (UMEs) regulate inflammation by regulating ubiquitination and methylation (Tarcic et al., 2016; Zhao et al., 2020), which may also regulate inflammation in tumor cell development. In addition, Li R. J. et al. (2015) revealed that HSP27 (Kostenko and Moens, 2009) (a heat shock protein closely associated with malignant tumors) phosphorylation can activate the p38 MAPK pathway to regulate protein kinase B, which in turn promotes glioblastoma cells and produces an inhibitory effect on apoptosis.

## 4 Effects of polysaccharides on tumors

**FIGURE 2:**
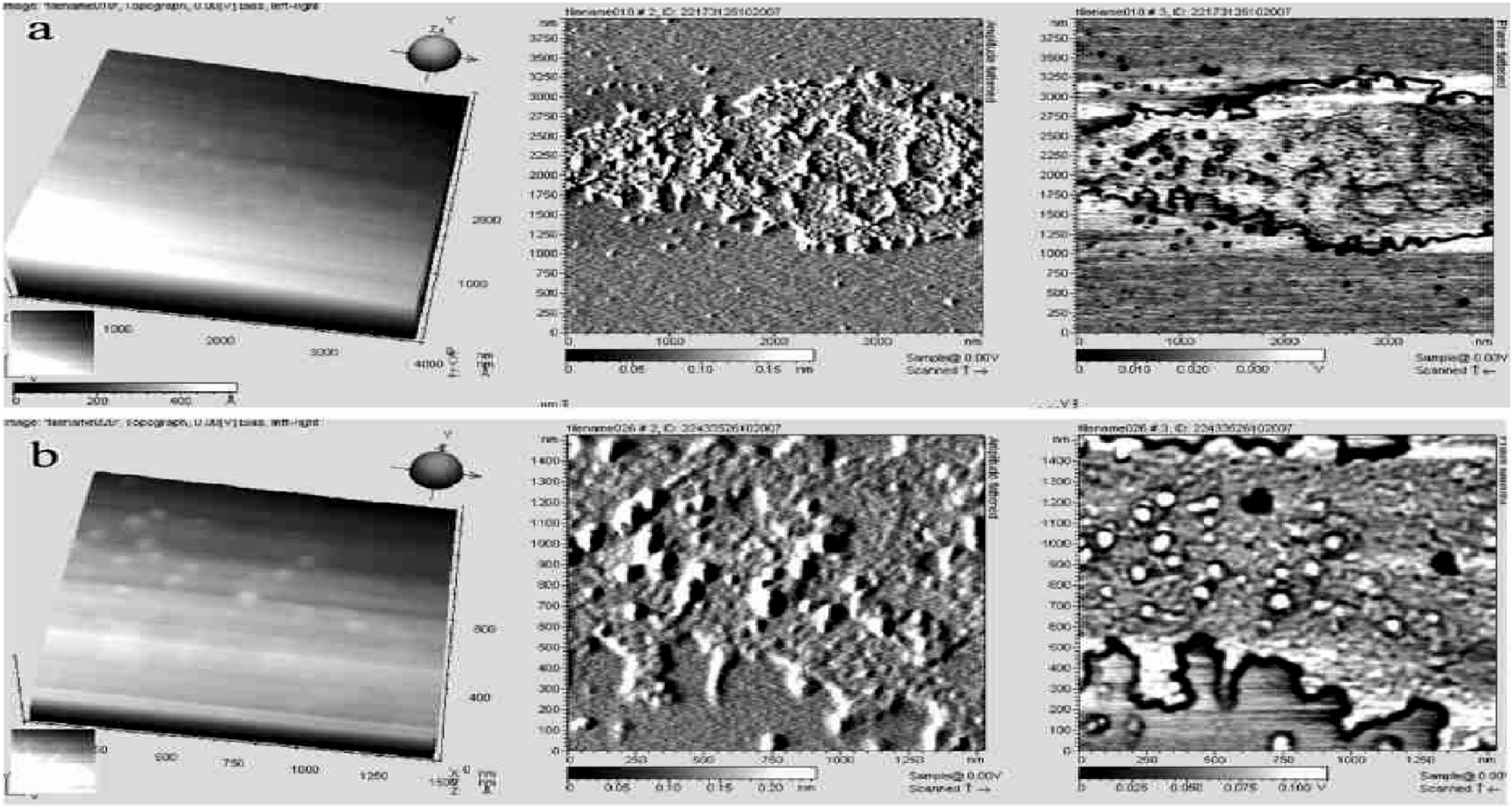
The AFM picture of green tea amylase. **(a)** 4⋅2 μm × 4⋅2 μm; **(b)** 1⋅5μm × 1⋅5 μm (Qin et al., 2009).

Plant polysaccharides have been shown to have a variety of medicinal activities, such as inhibiting cancer cell proliferation (Bae et al., 2020; Gutierrez-Castañeda et al., 2020), inducing apoptosis of tumor cells (Ren et al., 2021) and improving immune activity against tumors (Xie et al., 2020) et al. Its chemical composition and biological activity are also closely related to tumor treatment, and it has significant inhibitory effect on the activities of tumors in the human body (Figure 2).

### 4.1 Extraction of plant polysaccharides

Plant polysaccharides separation and purification system has evolved with research demands and industrial applications, aiming at maximize structural integrity while achieving efficient extraction. Traditional hot water extraction (HWE) disrupts plant cell walls via high temperatures (90°C–100°C) and is suitable for thermal stable polysaccharides such as starch and cellulose. However, it has disadvantages such as long processing time, low yields, and co-extraction of impurities. For instance, the extraction yield of olive leaf polysaccharides is only 7.2% by HWE method (Khemakhem et al., 2018; Huang and Huang, 2020; Ahmad et al., 2022). Alkali extraction enhances efficiency by cleaving ester bonds in acidic polysaccharides (e.g., pectin, uronic acids). But strong alkaline conditions may lead to t risks β-elimination of glycosidic bonds, so it is necessary to strict control the pH to preserve structural integrity (Kuroda et al., 2014; Huang and Huang, 2020). Emerging physical-assisted technologies have gained prominence. Microwave-assisted extraction (MAE) increases yield within minutes by taking advantage of the molecular polarization effect. For example, phenolic content in citrus leaf polysaccharides via MAE was 4.5-fold higher than HWE (Mohammadi et al., 2024). Ultrasound-assisted extraction (UAE) mechanically disrupts cell walls through cavitation. Taking Allium chinense epidermal polysaccharides as an example, its yield reaches 30.08% (30.1% higher than HWE), and energy consumption is reduced by 77.3% (Li et al., 2025). However, excessive microwave or ultrasonic power may fragment polysaccharide chains, parameter optimization is needed to balance efficiency and structural preservation (Li et al., 2025).

Novel biotechnological approaches demonstrate unique advantages. Supercritical fluid extraction (SFE) employs supercritical CO_2_to selectively extract non-polar polysaccharides under mild conditions, avoiding thermal degradation and enabling solvent recovery, despite the high equipment costs (Zou et al., 2018). Enzymatic-assisted extraction (EAE) utilizes cellulases or pectinases to hydrolyze cell wall components, improving yields by >50% over HWE under gentle conditions, aithough enzyme expenses limit its scalability (Huang and Huang, 2020). Probiotic fermentation enhances polysaccharide bioactivity via microbial secretion of organic acids and enzymes, but requires extended fermentation cycles (Wang et al., 2024).

Pressurization technologies like pulsed electric field (PEF) and pressurized liquid extraction (PLE) are increasingly being adopted for special applications. PEF induces cell membrane electroporation for low-temperature extraction of thermolabile polysaccharides, while PLE accelerates solvent penetration under high pressure (Ahmad et al., 2022; Li et al., 2025). Ultrafiltration enables molecular weight-based fractionation but faces challenges in membrane fouling (Ahmad et al., 2022).

Current research emphasizes multi-technology integration (e.g., enzyme-ultrasound synergy (Wang et al., 2024), microwave-supercritical coupling (Huang et al., 2024) to overcome the limitations of single methods. Future advancements must align with green chemistry principles and industrial feasibility to promote precise applications in functional foods and drug delivery systems. Table 1 shows a comparison of the main separation and extraction methods of plant polysaccharides.

**TABLE 1:** Comparison of major isolation and extraction methods for plant polysaccharides.

Methods	Application	Advantage	Disadvantages	References
Hot water extraction (HWE)	Heat stabilized polysaccharides (cellulose, starch)	Low cost, simple operation, no solvent pollution	Low efficiency, time-consuming, impurity co-extraction	Huang and Huang (2020)
Alkali extraction	Acidic polysaccharides (e.g., pectin, glucuronides)	High yield and good selectivity	Risk of glycosidic bond breakage, need for neutralization, environmental contamination	Kuroda et al. (2014), Huang and Huang (2020)
Microwave-assisted extraction	Rapid extraction (citrus leaf phenolics)	Short time, high yield, low energy consumption	Heat-sensitive polysaccharides are susceptible to degradation and high equipment costs	Ahmad et al. (2022), Mohammadi et al. (2024)
Ultrasound-assisted extraction (UAE)	Cell wall dense material	Significant efficiency improvement, green energy saving	High power leads to chain breakage, parameter optimization required	Ahmad et al. (2022), Li et al. (2025)
Supercritical Fluid Extraction (SFE)	Non-polar polysaccharides (e.g., fat-soluble components)	Low temperature, high efficiency, non-toxic solvent, good selectivity	Expensive equipment, high CO_2_ purity requirements	Zou et al. (2018)
Enzymatic hydrolysis	Complex substrates (e.g., lignocellulose)	Moderate conditions, high specificity, significant yield improvement	High enzyme cost and substrate specificity	Huang and Huang (2020)
Probiotics fermentation	High value-added active polysaccharides	Synergistic increase in yield and activity, environmentally friendly	Long cycle time and complex screening	Wang et al. (2024)
High-voltage pulsed electric field (PEF)	Heat-sensitive polysaccharides (e.g., beta-glucan)	Low temperature and high efficiency, low energy consumption, no thermal damage	Equipment specialization, electrode loss	Ahmad et al. (2022), Li et al. (2025)

### 4.2 Chemical structure and biological activity of polysaccharides

A polysaccharide is a macromolecular substance comprising more than 10 monosaccharide molecules linked by glycosidic bonds. The complex structure of polysaccharides is directly related to biological activity, and most polysaccharides with a β-helical structure have strong biological functions (Yang et al., 2023). Molecular weight is related to the biological activity of polysaccharides, and is considered to be one of the important parameters for measuring the anti-tumor activity of polysaccharides. Some studies have shown that high molecular weight dextrans have more potent anti-tumor activity than low molecular weight dextrans (Barbosa and Junior, 2020). While Liu et al. (2023) studied sour jujube polysaccharides and found that polysaccharides with low molecular weight have higher antioxidant activity. Pei et al. (2022) found that the low molecular weight fractions of honeysuckle polysaccharide extracts had high hypoglycemic bioactivity. It was also found that arabinoxylan extracted from wheat bran with low molecular weight has better antidiabetic activity (Xie et al., 2016; Lv et al., 2021). Wu et al. (2021) discussed the polysaccharides of sour pulp plants and concluded that the polysaccharides of sour pulp plants are mostly heteropolysaccharides, and the main composition of their monosaccharides includes L-rhamnose monohydrate and arabinoxylan, which showed more obvious antioxidant activities. Xie et al. (2023) found that the more complex the composition of monosaccharides is, the better the biological activity of polysaccharides is, and concluded that the polysaccharides of edible and medicinal plants with hypoglycemic activity mostly contain Gal, Glu, Ara, and Man. The polysaccharide extracts extracted under different extraction methods, even if the types of monosaccharides are the same, have significant differences in proportion, which will also affect the biological activity of polysaccharides (Ross et al., 2015). Li et al. (2020) confirmed that the chemical composition and bioactivity of the polysaccharide extracts of Orthorhombic mushrooms are highly correlated with their mode of extraction and less correlated with their extraction site.

Many studies have demonstrated that polysaccharides contain a wide range of biological activities that are beneficial to the human body. Some lower plants have many polysaccharide compounds, such as lichen polysaccharides, which are almost unique to lichens, and more than 80% of lichens contain this class of polysaccharides and exhibit high antitumor activity. Yu et al. (2019) extracted ASP4 from Astragalus polysaccharides with high Glep content, showing vigorous antitumor activity. Generally, polysaccharides exert antioxidant activity by scavenging excess reactive oxygen species, eliminating reactive free radicals and increasing antioxidant enzyme activity. Chen and Huang (2019) found that cucumber polysaccharides have strong OH radical scavenging ability and the ability to reduce iron ions. The polysaccharides of Platycodon grandiflorum are also reported to have apparent antioxidant activity (Li et al., 2023). The hypoglycemic mechanism of polysaccharides is often in the form of protecting pancreatic β-cells, promoting insulin secretion, and improving glucose metabolism. Li etc. (Li Q. et al., 2017) compared the hypoglycemic activity of yam polysaccharides with different molecular weights and different concentrations, and found that the lower molecular weight and the higher concentration have higher the hypoglycemic activity. Guo et al. (2017) demonstrated that the polysaccharides of the brook lampoon persistent calyx can promote insulin secretion and hypoglycaemic activity in the mice.

In addition to the prominent biological activities of antitumour, antioxidant, and anti-glycemic, polysaccharides have a wide range of biological activities, such as regulating intestinal flora (Wei et al., 2023), anti-inflammatory activity (Tong et al., 2011), and immunomodulatory activity (Fan et al., 2016), etc. Although the bioactivities of polysaccharides have been recognized to some extent, many aspects still need to be further investigated.

Future studies can explore the intrinsic connection between the chemical structure of polysaccharides and various biological activities in greater depth. The mechanism of the biological activities of polysaccharides can be further revealed by refining the analysis from the more subtle conformational levels such as molecular weight, monosaccharide composition and molar ratio. Meanwhile, the relationship between polysaccharide structure and biological activity can be further clarified by synthesizing polysaccharides of different structures and comparing their biological activity differences. Table 2 lists some of the popular polysaccharide components and related information.

**TABLE 2:** Some popular polysaccharide fractions and related information.

Category	Molecular weight	Composed of monosaccharides (proportion)	Main biological activity	References
CY-2	2.69 × 10^4^Da	Glu:Man (33.5: 56.9)	Lowers blood sugar	Tian et al. (2023)
GLSP	8.20 × 10^4^Da	Ara:Glc:Gal (1.23:90.82:7.95)	Anti-inflammatory	Wen et al. (2022)
WSG	1.00 × 10^6^Da	Glu:Gal:Man:GlcN:Fuc (20:4:4:3:2)	Anti-tumor	Hsu et al. (2020)
TFLP-1	7.58 × 10^4^Da	It is mainly composed of mannose	Antioxidant	Lee et al. (2023)
LPNP	1.16 × 10^4^Da	Xyl:GalA:Gal:Glu:Rha:Ara	Antioxidant Anti-inflammatory	Zheng et al. (2023)
HLP	7.34 × 10^4^Da	Glu:Rha:Gal:Man (4.58:0.46:8.51:10.25)	Anti-tumor	Ge et al. (2022)
LMW-BSP	2.30 × 10^4^Da	Glu:Man (1.00:1.26)	Anti-tumor	Liu et al. (2022)
Cordyceps sinensis polysaccharides	2.80 × 10^4^Da	Ara:GalA:Man:Glc: Gal (1.00:2.26:7.43:8.68:10.90)	Anti-tumor	(Qi et al., 2020)
Brazil mushroom polysaccharides	7.40 × 10^4^Da	Man:Ara:Glc:Xyl:Rib:Gal (1.00:1.12:2.16: 3.56:4.81:26.44)	Anti-tumor	Zhou and Chen (2011)
Boletu edulis polysacchardes	1.13 × 10^4^Da	Rha:Ara:Glc: Gal (1.00:1.23:2.23:2.46)	Anti-tumor	(Wang et al., 2014)

### 4.3 Effect of polysaccharides on tumor cell proliferation, apoptosis, metastasis and invasion *in vivo*

Because cancer is listed as one of the most popular diseases in the 21st century (Sung et al., 2021), the anti-tumor activity of polysaccharides is the hotspot. Taking advantage of the anti-tumor activity of polysaccharides and their small side effects on the human body to assist in tumor treatment has become a new therapeutic trend for tumors. Under normal circumstances, cells in the human body will undergo normal proliferation, differentiation and apoptosis, while mutated tumor cells will proliferate abnormally and endanger human health. Some studies have shown that polysaccharides inhibit the proliferation of tumor cells in the human body. Bamodu et al. (2019) found that baicalin polysaccharides extracted from Scutellaria baicalensis significantly inhibited the growth of tumors in mice with non-small cell carcinoma (NSCLC), and had a significant inhibitory effect on the enlargement of this type of cancer cells. Cordyceps polysaccharides have also been shown to inhibit the proliferation of colon cancer cells HCT116 (Abu-Khudir et al., 2020). A large number of studies have confirmed that polysaccharides can play a role in inhibiting the proliferation of tumor cells *in vivo*, and the effect of this bioactivity is significant. But at present, the mechanism that polysaccharides direct inhibit tumor cell proliferation has not yet been elucidated thoroughly, and further research into the core mechanism is needed.

Polysaccharides can also achieve anti-tumor effects by promoting apoptosis of tumor cells. Corso et al. (2021) and others reviewed the anti-breast cancer effect of polysaccharides and found that most of the polysaccharides can effectively promote apoptosis of breast cancer cells. Angelica polysaccharides can directly act on Gal-3 to achieve the efficacy of promoting apoptosis of cancer cells (Liu W. J. et al., 2020). Combined with the analysis of existing literature, it was found that polysaccharides usually induce apoptosis through endogenous and exogenous pathways. The exogenous pathway, also known as the cytoplasmic pathway, is triggered by the Fas death receptor (a member of the TNF receptor superfamily of tumor necrosis factors). The endogenous pathway or the mitochondrial pathway, releases cytochrome C from mitochondria when stimulated and activates death signalling. Both path will activate cysteine aspartate proteases that cleave regulatory and structural molecules, ultimately leading to cell death (Ghobrial et al., 2005), More plant polysaccharides can be further investigated for their apoptosis-promoting mechanisms in tumor cells, and the potential of polysaccharides as antitumor agents can be explored.

The metastatic and invasive ability reflects the degree of malignancy of the tumor. And the inhibitory effect on them reflects the anti-tumor activity of polysaccharides. Zhong K. et al. (2020) demonstrated that Araucaria lactiflora polysaccharides (ALP) would inhibit metastasis and invasion of human osteosarcoma U-2OS. Charcot polysaccharides inhibit the expression of basic fibroblast growth factor, which in turn inhibits the growth and migration of breast cancer CFAs (Hao et al., 2016). Another study showed that hyssop polysaccharides could inhibit lung cancer metastasis by suppressing the expression of the invasion-related molecule matrix metalloenzyme (Zhong C. L. et al., 2020). The effectiveness of polysaccharides in inhibiting metastasis and invasion of tumor cells has been confirmed in many domestic and international literatures, and in general, polysaccharides inhibit outward metastasis and invasion of tumor cells by affecting the expression of factors related to metastasis or invasion.

**FIGURE 3:**
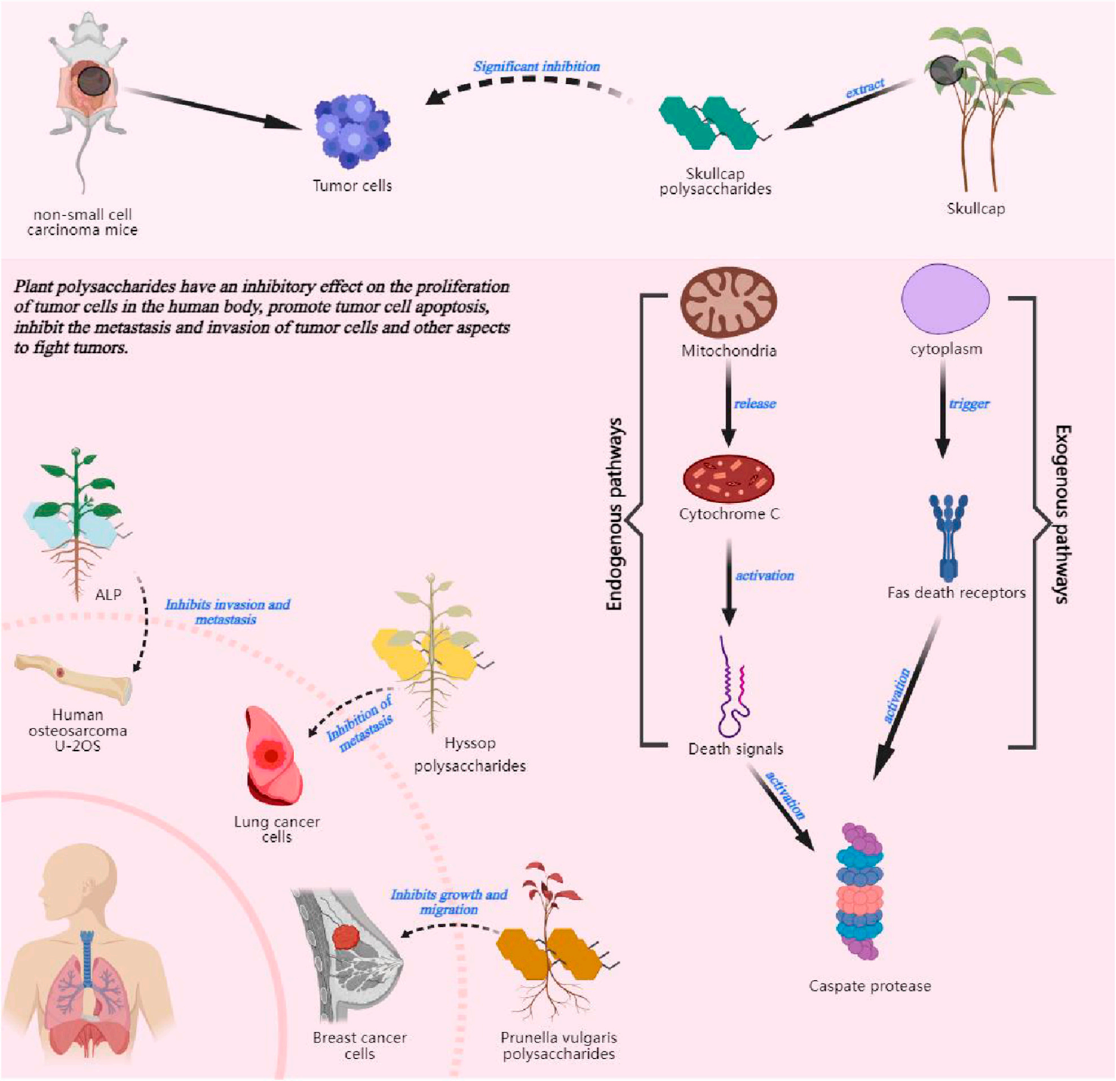
The effect of plant polysaccharides on epigenetic effects on tumors.

In summary, polysaccharides have inhibitory effects on the proliferation of tumor cells in the human body. Polysaccharides can promote apoptosis of tumor cells, inhibit the metastasis and invasion of tumor cells and other aspects of anti-tumor, and can achieve a more ideal anti-tumor efficacy. Polysaccharides are some of the most significant extracts with anti-tumor activity in Chinese herbal medicines at present. However, in the field of plant polysaccharides with anti-tumor bioactivity, most of the relevant articles focus on the mechanism of polysaccharide inhibition of tumor, and the chemical properties of polysaccharides remain in the preliminary stage of crude polysaccharide extraction methods and the determination of physicochemical properties. The use of non-homogeneous polysaccharides as the study object may result in the presence of other compounds such as lipopolysaccharide, polyphenols or proteins, and so on, which may affect the determination of biological activity. Therefore, there is an urgent need to study homogeneous polysaccharides and further explore their anti-tumor bioactivity and intrinsic mechanism. The possible future research work is shown in Figure 3.

## 5 Effects of polysaccharides on the genetic regulation of performance

### 5.1 Interaction of polysaccharides with epigenetic regulation

In nature, polysaccharides are present in almost all organisms, including seed tissues, herbaceous stems and leaves (Li J. et al., 2017) Polysaccharides are essential components of the organism, which have critical pharmacological activities against tumors, and therefore have a significant value of potential tumor therapeutics to be explored. Combined with the epigenetic regulation of tumors, there is also a corresponding mechanism for the role of polysaccharides and epigenetics.

In previous studies, translocated dioxygenase 2 (TET2) was found to play a key role in the pathological mechanism of apoptosis in diabetic neurons (Liu R. et al., 2020; He et al., 2022), TET2 is a key enzyme in DNA demethylation, which catalyzes the oxidation of 5-methylcytosine (5 mC) to 5-hydroxymethylcytosine (5hmC), 5-formyl cytosine (5 fC) and 5-carboxylcytosine (5caC), therefore altering the epigenetic status (Chen L. et al., 2023). In contrast, it has been shown that Dendrobium polysaccharide (PD) enhances TET2 protein stability and promotes demethylation by activating phosphorylated AMRK in the mouse cerebral cortex induced by a high-fat diet (HFD), and it was predicted that DP may contribute to TET2 enzyme activity by improving tricarboxylic acid cycle (TCA cycle) homeostasis (Wu and Zhang, 2017). In addition, an animal model of induced intratumoural hypoxia in mice demonstrated that basil polysaccharides could inhibit the expression of histone modifying enzymes JDJM1A, JDJM2B in a dose-dependent manner, suggesting that basil polysaccharides could inhibit the growth of relevant tumors through the modulation of histone demethylating enzymes. Meanwhile, Toubhans et al. (2023) showed that inorganic selenium nanoparticles of chitosan, which naturally produces alkaline polysaccharides, contribute to the regulation of histone methylation in an ovarian cancer cell model, and that the trace element selenium plays a key role in the redox reaction through the incorporation of selenocysteine in antioxidant enzymes. It has also been shown that Ganoderma lucidum polysaccharides can inhibit the progression of hepatocellular carcinoma by inducing phosphorylation activation of the MAPK and NF-KB signalling pathways and regulating macrophage polarisation. Yan et al. (2018) investigated Cistanches polysaccharides (CDPS) and found that it increased BDNF (Ji et al., 2014; Leal et al., 2014) (brain-derived neurotrophic factor, an essential neurotrophic factor for neuronal and neuroglial important neurotrophic factor, considered as one of the key proteins in memory formation) expression, thus improving and promoting neuronal survival in favor of Alzheimer’s disease prevention (Lin et al., 2017). Earlier studies showed that lysosome production in tumor cells is regulated by histone acetylation (Li X. J. et al., 2017), which is subordinate to epigenetic histone modifications (Chen H. L. et al., 2023). H3K27ac is a widely studied histone acetylation modification that is generally catalyzed by histone acetyltransferases (HAT) EP300 and CBP (Durbin et al., 2022). Another study showed that tea tree mushroom polysaccharide (ACP) significantly caused lysosomal over-accumulation and enhanced the acid value in HCT-116 cells (rectal cancer cells), resulting in lysosomal dysfunction. ACP significantly upregulated H3K27ac protein expression and increased the expression of acetyltransferase CBP, confirming that tea tree mushroom polysaccharide can inhibit rectal cancer cells’ proliferation (Peng et al., 2022).

In conclusion, the regulation of epigenetic inheritance by polysaccharides is usually achieved through the activation of induced phosphorylation, inhibition of the expression level of histones associated with tumor development, induction of demethylation and activation of related signalling pathways. These regulatory pathways can, to a certain extent, produce an inhibitory effect on the occurrence and development of tumor cells. Further study on the relationship between polysaccharides and epigenetic inheritance, and may be able to promote treatment of tumors or other diseases associated with epigenetic inheritance.

### 5.2 Effect of polysaccharides on the regulation of miRNA expression

MiRNA (micro RNA) belongs to a class of ncRNA (non-coding RNA) with a length of less than 200 nucleotides. We comprehensively analyzed the relevant literature in recent years and discussed the effects of polysaccharides on the regulation of microRNA.

Some studies have shown that mir-139-5p (belonging to the miRNA family) is downregulated in expression in a variety of tumor tissues and can function as a potential tumor suppressor (You et al., 2019), Poria cocos polysaccharides can significantly change the expression of miRNAs and inhibit lung cancer cells in animal models (Zhang et al., 2021). Guo et al. speculated that the inhibition of human papillary thyroid cancer cells by Astragalus polysaccharides might be related to reduction of novel-miR-26 expression and elevation of FOXO1 (an oncogene) protein expression to achieve inhibition of tumor cell proliferation (Guo et al., 2023). Polysaccharides contained in compound astragalus and danshen extracts can effectively inhibit chemically induced hepatocarcinogenesis and tumor growth in an animal model of related miRNA mice (Wu et al., 2019). Li et al. (2015) showed that miR-125 was significantly upregulated at the level of intra-tumor regulatory T cells in hepatocellular carcinoma mice injected with Ganoderma lucidum polysaccharides, and that Ganoderma lucidum polysaccharides dose-dependently reduced the expression of oncogenes. It has also been shown that Ganoderma lucidum polysaccharides caused upregulation of miR-3131 expression in HepG2 cells (Shen et al., 2014), suggesting that Ganoderma lucidum polysaccharides promote miRNA expression to achieve tumor cell suppression. Guo et al. (2020) found that Astragali polysaccharides increased FBXW7 levels in OV-90 cells through the blockade of miR-27a-mediated targeting of FBXW7 to exert its anti-tumor effect.

Polysaccharides can achieve tumor suppression by regulating miRNA expression, and this effect has been verified in animal models (Li C. et al., 2015). Several categories of the miRNA family are downregulated in various tumor tissues, and may function as potential tumor suppressors. Plant polysaccharides such as Poria cocos and Ganoderma lucidum polysaccharides have also been demonstrated in several studies to regulate the spread of tumor cells by reducing the expression of relevant miRNAs, increasing the expression of oncogenes, and modulating the upregulation of immune cell levels. Future studies can further explore the role of miRNAs in tumor development and treatment. The effects of different polysaccharides on miRNA expression and their regulation of tumor cell proliferation and metastasis can be further investigated. Findings in these areas will provide new directions and strategies for potential therapeutic approaches for tumor treatment and prevention.

## 6 Other developmental applications of polysaccharides

In recent years, a large number of studies have shown that the use of traditional Chinese medicine polysaccharides as adjuvant drugs in combination with chemotherapeutic drugs can achieve the effects of inhibiting tumor growth, improving the body’s immunity, regulating the body’s internal environment, mitigating the adverse effects, improving the quality of survival and cycle of the patients, and increasing the efficacy and reducing the toxicity (Liu et al., 2019). Polysaccharides are also frequently applied in clinical practice for the combined treatment of tumors. It is well known that fluorouracil can inhibit the index of immune organs. Tang et al. (2012) found that 5-fluorouracil could reduce the indexes of spleen and thymus, while these indexes were elevated after combined use of LBP polysaccharides, which demonstrated the ability of LBP to improve the immune function of the organism, and to enhance the anti-tumor ability of the organism. In addition, polysaccharides can also be combined with CTX (Song et al., 2010) (a broad-spectrum anticancer drug). Combined use of cornhusk polysaccharides and CTX could enhance the antitumor efficacy of CTX, as well as improve the quality of survival of H22 loaded mice, reduce the adverse damage of CTX to immune organs, and alleviate the side effects caused by chemotherapy (Xian-Chuang et al., 2014). Ginseng polysaccharides can also be used in combination with CTX to improve the immune damage caused by chemotherapy, and enhance the immunity of animal models, suggesting that ginseng polysaccharides have the effect of improving the immunity of the organism in tumor chemotherapy (Jia et al., 2013).

In addition to participating in immune regulation by combining with anticancer drugs for tumor treatment, plant polysaccharides, there are other efficacies involved in combined therapy. Some studies have proved that plant polysaccharides have a protective effect on the organs. Combined use of 5-fluorouracil and ashwagandha polysaccharides can improve the liver index and reduce chemotherapy damage to the liver (Mao et al., 2019). LBP polysaccharides are also able to reduce the toxicity of chemotherapeutic drugs on cells and increase the activity of kidney cells, thus accelerating the excretion of toxins in the organism, reducing the adverse effects of chemotherapy, and to a certain extent playing a protective role for the kidneys (Bie et al., 2015). In addition, there are other studies have shown that polysaccharides can participate in the treatment by inhibiting the formation of new blood vessels in tumors. Mushroom polysaccharides cisplatin drug can promote apoptosis of tumor cells and inhibit tumor vascular neogenesis, which significantly improves the remote efficacy of the treatment (Liu and Lin, 2018). Chen (Chen et al., 2018) et al. found that combined use of Ganoderma lucidum polysaccharides and cisplatin can increase the inhibition. effects of cisplatin on the growth of tumors and angiogenesis in T24 nude mice.

In summary, plant polysaccharides are now gradually applied to clinical participation in the treatment of tumors, with the effects of human organ protection, inhibition of tumor neoangiogenesis and other aspects. Existing research showed good clinical effect of plant polysaccharides. Plant polysaccharides can promote the antitumor drugs absorption and reduce the damage caused by the drugs, improve the survival cycle and quality of the target cells and tissues, and increase the effect of antitumor treatment.

**FIGURE 4:**
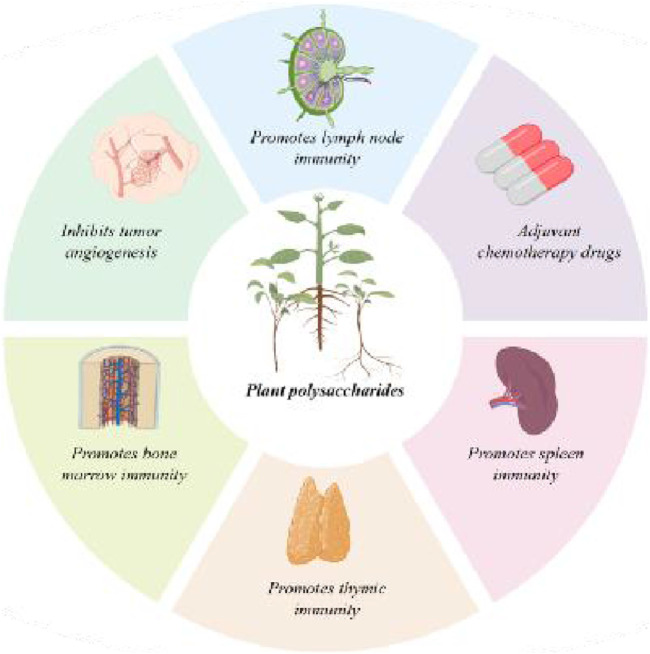
Diagram of other applications of plant polysaccharides.

As a potential drug for the treatment of tumors, other positive effects of plant polysaccharides for tumor treatment is worth further study (Figure 4).

## 7 Conclusion and outlook

A lot of research has been carried out in plant polysaccharides, targeting the properties of their broader antitumor pharmacological value. Clinical trials with different polysaccharides have proved the tumor chemopreventive effects of these macromolecules. These findings require further investigation to determine the pharmacokinetic and pharmacodynamic aspects. Although many potential mechanisms have been investigated, most studies have been conducted in animal models and at the cellular level. The effects of drugs on animals cannot be fully extrapolated to humans due to the differences in physiological structures between animals and humans. Furthermore, because polysaccharides are large molecular compounds, they are difficult to enter cells in their natural form and usually act through metabolic pathways in the body. Therefore more attention is need in clinical research and application of polysaccharides.

Currently, polysaccharides have a surprising performance in clinical applications. Its combined application with chemotherapeutic drugs reduces the toxins produced by chemotherapy, reduces drug resistance and improves the therapeutic efficacy. Some other benefits are equally helpful to the patient’s healing, protecting organs and inhibiting tumor new vessels formation. In addition, because of the uniqueness of the polysaccharide structure and its pharmacological properties, it can be used as a drug or drug carrier to target the treatment of tumors and play an anti-tumor role, which is also in line with the trend of multi-targeting in tumor treatment and the concept of precision medicine.

This review shows that the mechanism of polysaccharides on epigenetic regulation has become a research hotspot in recent years. More studies have shown that the effect of polysaccharides on epigenetics can be applied to anti-tumor. Polysaccharides regulate the activity of certain DNA methylation enzymes, histone modifying enzymes, promote phosphorylation and ubiquitylation, and enhance the activity of specific pathways, and then inhibit the various related activities of the tumor cells. These effects suggest that polysaccharides can not only be used as an adjunctive therapeutic option to anti-tumor drugs, but also has the potential to become the mainstream therapeutic strategy. The existing studies have mainly focused on plant polysaccharides, such as Rhizoma Polygonati Odorati, Ganoderma Lucidum, Cistanche, etc. Relatively few studies have been conducted on other species of polysaccharides, such as animals and fungi, Therefore the effects of polysaccharide extracts on tumor cells through the modulation of epigenetic inheritance have not yet been comprehensively investigated. Thus, it is an opportunity and a challenge to further study the role of plant polysaccharides on epigenetic regulation in tumors and to develop new clinically applicable products. It is believed that with the in-depth related research, there will be a breakthrough in the regulation of tumor treatment by plant polysaccharides through epigenetic mechanisms in the near future.
